# Distinct motor impairments of dopamine D1 and D2 receptor knockout mice revealed by three types of motor behavior

**DOI:** 10.3389/fnint.2014.00056

**Published:** 2014-07-15

**Authors:** Toru Nakamura, Asako Sato, Takashi Kitsukawa, Toshihiko Momiyama, Tetsuo Yamamori, Toshikuni Sasaoka

**Affiliations:** ^1^Division of Brain Biology, National Institute for Basic BiologyOkazaki, Japan; ^2^Department of Basic Biology, Graduate University for Advanced Studies (SOKENDAI)Okazaki, Japan; ^3^Laboratory of Neurochemistry, National Institute for Basic BiologyOkazaki, Japan; ^4^Department of Laboratory Animal Science, Kitasato University School of MedicineSagamihara, Japan; ^5^KOKORO-Biology Group, Laboratories for Integrated Biology, Graduate School of Frontier Biosciences, Osaka UniversityOsaka, Japan; ^6^Department of Pharmacology, Jikei University School of MedicineTokyo, Japan; ^7^Department of Comparative and Experimental Medicine, Brain Research Institute, Niigata UniversityNiigata, Japan

**Keywords:** dopamine receptor, congenic knockout mice, motor activity, skilled motor performance, behavioral task, rota-rod, Step-Wheel system

## Abstract

Both D1R and D2R knock out (KO) mice of the major dopamine receptors show significant motor impairments. However, there are some discrepant reports, which may be due to the differences in genetic background and experimental procedures. In addition, only few studies directly compared the motor performance of D1R and D2R KO mice. In this paper, we examined the behavioral difference among N10 congenic D1R and D2R KO, and wild type (WT) mice. First, we examined spontaneous motor activity in the home cage environment for consecutive 5 days. Second, we examined motor performance using the rota-rod task, a standard motor task in rodents. Third, we examined motor ability with the Step-Wheel task in which mice were trained to run in a motor-driven turning wheel adjusting their steps on foothold pegs to drink water. The results showed clear differences among the mice of three genotypes in three different types of behavior. In monitoring spontaneous motor activities, D1R and D2R KO mice showed higher and lower 24 h activities, respectively, than WT mice. In the rota-rod tasks, at a low speed, D1R KO mice showed poor performance but later improved, whereas D2R KO mice showed a good performance at early days without further improvement. When first subjected to a high speed task, the D2R KO mice showed poorer rota-rod performance at a low speed than the D1R KO mice. In the Step-Wheel task, across daily sessions, D2R KO mice increased the duration that mice run sufficiently close to the spout to drink water, and decreased time to touch the floor due to missing the peg steps and number of times the wheel was stopped, which performance was much better than that of D1R KO mice. These incongruent results between the two tasks for D1R and D2R KO mice may be due to the differences in the motivation for the rota-rod and Step-Wheel tasks, aversion- and reward-driven, respectively. The Step-Wheel system may become a useful tool for assessing the motor ability of WT and mutant mice.

## Introduction

Dopamine plays a critical role in a number of brain functions in the nervous systems. In mammalian brains, dopamine affects motor control, feeding behavior, cognitive functions, and habits, and is involved in mediating the reactivity of the organism to the environment (Moore and Bloom, 1978; Gingrich and Caron, 1993; Schultz, 2007; Grahn et al., 2008; Graybiel, 2008; Kim et al., 2010). Dopamine signals are mediated by dopamine receptors (DRs) of five subtypes and are classified as D1-like receptors (D1R, D5R) and D2-like receptors (D2R, D3R, D4R). D1R activates adenylyl cyclase, whereas D2R inhibits it and increases potassium channel activity. D1R is highly expressed in the striatum (caudate and putamen), nucleus accumbens, olfactory tubercle, cerebral cortex and amygdala, and is widely distributed. D2R is highly expressed in the striatum, olfactory tubercle, nucleus accumbens, substantia nigra pars compacta (SNc) and ventral tegmental area; these last two nuclei give rise to the major dopaminergic tracts (Gingrich and Caron, 1993; Vallone et al., 2000). One of the main targets of dopamine related to motor control is the striatum.

The striatum is a major input component of the basal ganglia, which plays critical roles in various brain functions including motor control and motor learning (Graybiel, 1995; Reynolds and Wickens, 2000). The inputs to the striatum come from many regions of the cortex and SNc, and the inhibitory outputs to other basal ganglia nuclei form modulatory pathways. The neurons projecting from the striatum are GABAergic medium-sized spiny neurons (MSNs), which can be subdivided into two sub-populations, the striatonigral and striatopallidal neurons (Gerfen et al., 1990; Schiffmann et al., 2007). These MSNs constitute two pathways of the basal ganglia. The striatonigral MSNs directly project to the output part of the basal ganglia, the internal segment of the globus pallidus (GPi) and the substantia nigra pars reticulata (SNr) (direct pathway). The striatopallidal MSNs indirectly project to GPi/SNr (indirect pathway) via the external segment of the globus pallidus (GPe) and subthalamic nucleus. According to the classical model, these two pathways counteract each other, cooperating to maintain a balance of movements; the direct pathway facilitates and the indirect pathway inhibits motor activity (Albin et al., 1989; Alexander and Crutcher, 1990; Gerfen et al., 1990; Graybiel, 1995; Gerfen, 2000; Reynolds and Wickens, 2000). Interestingly, these two types of counteracting MSN, the striatonigral and striatopallidal MSNs, express different dopamine receptors, D1R and D2R, respectively (Schiffmann and Vanderhaeghen, 1993). Furthermore, D1R and D2R exert opposite effects, as mentioned above. Thus, it would be important to clarify how dopamine affects on motor behaviors through these counteracting receptors by directly comparing D1R and D2R knockout (KO) mice.

D1R KO mice (Drago et al., 1994; Xu et al., 1994a,b; Smith et al., 1998; Centonze et al., 2003; Tran et al., 2005, 2008) and D2R KO mice (Baik et al., 1995; Yamaguchi et al., 1996; Kelly et al., 1997; Jung et al., 1999; Tran et al., 2002) provide indispensable information for understanding the functions of D1R and D2R. Nonetheless, studies on the spontaneous motor activity of D1R KO mice showed inconsistent results. This inconsistency may be due to the differences in the genetic background and experimental procedure used in such studies (Kelly et al., 1998; Fowler et al., 2002; McNamara et al., 2003; Holmes et al., 2004; Waddington et al., 2005). However, it is difficult to determine what exactly underlies such inconsistencies, given that different experimental procedures with different genetic backgrounds were employed in various studies. It is therefore important to compare the results obtained under the same experimental conditions and using the same procedure because a small difference can affect the animal behavior (Crawley, 1999; Wahlsten et al., 2003). In addition, motor ability and motor learning of congenic D1R and D2R KO mice have been reported (Kelly et al., 1998; Fowler et al., 2002; Wall et al., 2011) and a direct comparison between non-congenic D1R and D2R KO mice as well has been carried out (Kobayashi et al., 2004). However, no study on a detailed comparison between congenic D1R and D2R KO mice is available.

In this study, we used N10 congenic KO mice and compared them simultaneously under the same conditions. We monitored their motor activity in their home cages continuously for 5 days as normal spontaneous activity, in which the effects of external factors are negligible. For better understanding of characteristics of motor activity, we measured the intensity of activity and the duration and number of active and inactive bouts by examining the data of the motor activity in the home cage. Since DRs are involved in body weight gain and food consumption (Kim et al., 2010), we measured the volume of food and water intake while monitoring the motor activity. The direct and indirect pathways in the striatum exert opposite effects as reward systems; the direct pathway is critical for reward learning whereas the indirect pathway is critical for aversive learning (Hikida et al., 2010). Thus, we analyzed the motor ability of the KO mice using two motor behavior tasks, the rota-rod task and the Step-Wheel task, which are aversive- and reward-driven tasks, respectively.

## Materials and methods

### Animals

Mice lacking either D1R or D2R were generated in accordance with to the protocol previously published (Yamaguchi et al., 1996; Tran et al., 2002, 2005, 2008) and backcrossed for up to 10 generations with C57BL/6J (CLEA Japan Inc., Tokyo Japan) mice. Their genotypes were determined by PCR analysis of genomic DNA extracted from the tail of each mouse. As a control, C57BL/6J WT mice were purchased and kept at a constant temperature of 22 ± 2°C and humidity of 55 ± 5% with a 12 h light/dark cycle. All the experiments were performed in accordance with the guideline of the National Institutes of Health, and the Ministry of Education, Culture, Sports, Science, and Technology (MEXT) of Japan, and were approved by the Institutional Animal Care and Use Committee of the National Institutes of Natural Sciences and Kitasato University School of Medicine. We made all efforts to minimize the number of animals used and the incidence or severity of distress experienced by the animals.

### Spontaneous motor activity

Adult male mice (2–8 months old) were housed individually in a plastic cage under 12 h light/dark cycle (8:00–20:00) and fed with food (Rodent Diet CA-1, CLEA Inc., Tokyo, Japan) and water *ad libitum*. Before the start of measurement, we set the habituation time until 8:00 the following day. Spontaneous motor activity in home cage was measured using a pyroelectric infrared motion sensor above the cage (O'HARA & CO., LTD., Tokyo, Japan), as reported previously (Paemka et al., 2013). The sensor detects thermal radiation from the mouse and counts all movements including horizontal locomotion, rearing, and hanging on the lid. A computer program linked to the sensor recorded the cumulative activity count every 10 min. For the analysis of activity, the activity state was classified as follows on the basis of the number of movements: inactive state (0–9 counts/10 min), low- (10–199 counts/10 min), medium- (200–499 counts/10 min) and high-activity (over 500 counts/10 min) states, and the percentage of time spent in each state was calculated. For the analysis of duration and number of bouts, the inactive state and active states were classified as 0–9 counts/10 min and over 10 counts/10 min, respectively.

### Food and water intake

Food and water intakes were measured simultaneously with activity counts using a digital scale, which continuously weighed the food cup and the water bottle placed beside the cage (O'HARA & CO., LTD., Tokyo, Japan) as reported previously (Paemka et al., 2013). A computer program automatically recorded the weight change every 10 min. All the animal behaviors in the cage were recorded using a CCD camera and infrared illumination to confirm that the data properly reflected the animal behavior.

### Motor behavioral tasks

Adult male mice (6 months old at the beginning of the rota-rod task) were housed individually in plastic cages under 12 h light/dark cycle (9:00–21:00) and fed with food (Rodent Diet CE-2, CLEA Inc., Tokyo, Japan) and water *ad libitum*. To encourage food intake to maintain their health (Drago et al., 1994; McNamara et al., 2003), the D1R KO mice were given palatable food (Rodent Diet B-F, CLEA Inc., Tokyo, Japan) at the floor of their cages. The Rota-rod and Step-Wheel tasks were performed during the light phase (13:00–19:00). During the Step-Wheel task, the daily water intake was controlled.

### Rota-rod task

The rota-rod apparatus (MK-660A, MUROMACHI KIKAI CO., LTD., Tokyo, Japan) was used. The rod diameter was 3 cm. We used two different rotation speeds, 15 rpm (fast) and 5 rpm (slow), in this study. One trial was finished when any one of the following three events occurred. The mouse (1) fell, (2) remained up to 120 s, or (3) clung to the rod for two complete turns in which the mouse was considered to have fallen. Retention time (sec) was scored when one of the above mentioned events occurred. Three trials per day were performed and the interval between the trials was set at 30–60 s, during which time, the mice were put in their home cages. The presessions consisted of trials for three continuous days, during which the mice were placed on the stationary (0 rpm) rod for habituation to the apparatus. Following the presessions, the running sessions (for 7 consecutive days) was started. Between the groups of sessions, we set 1 day for rest, during which the mice spent their time normally in their home cages.

### Step-wheel system

**Figure 1:**
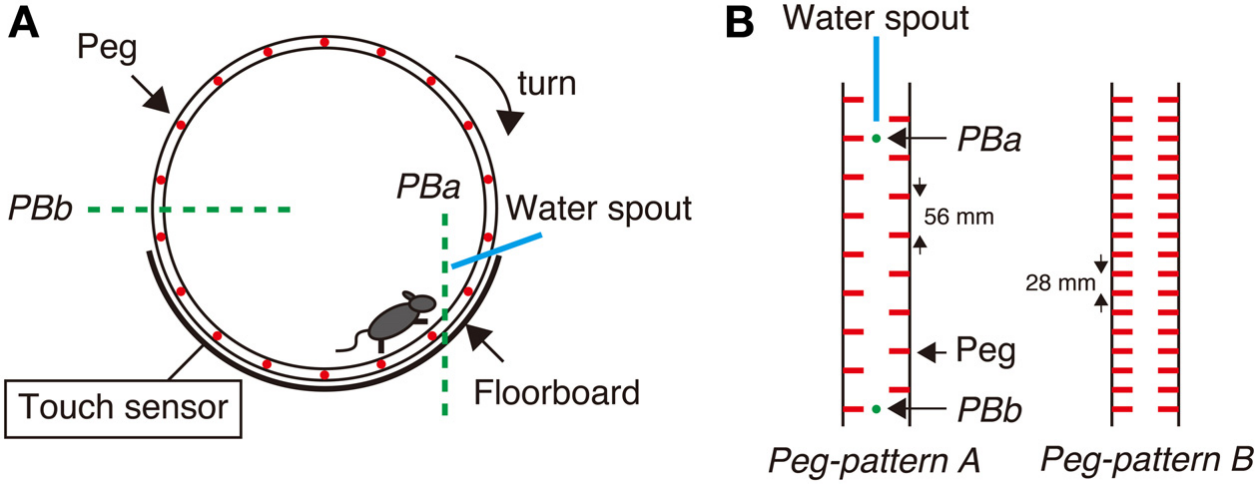
**Step-Wheel System. (A)** The wheel was driven by a motor, turning clockwise at a constant speed. The infrared photobeams (green dot lines) and an electric touch sensor were used for detecting mouse behaviors. *Photobeam a* (*PBa*) was used for detecting a mouse running in the vicinity of the spout; *Photobeam b* (*PBb*) was used for detecting the mouse when it was on the most downstream of the wheel. The touch sensor detected foot touches when the mouse fell down to the floorboard. **(B)** Two peg patterns used in this study. *Peg-pattern A* was designed for mice to run in the “walk” gait. *Peg-pattern B* was designed to have an easier and simpler pattern with twice as many pegs as *peg-pattern A*. The water spout and two photobeams (*PBa* and *PBb*) were set between the right and left pegs.

The Step-Wheel System (O'HARA & CO., LTD., Tokyo, Japan) was developed in our laboratory (Kitsukawa et al., 2011) (Figure 1). The wheel in this system is motor-driven and thus turns at a constant speed. Mice were trained to run on pegs in order to drink water as the reward. The alignment of the pegs was adjusted by experimenters. In this study we employed two types of simple peg patterns: *peg-patterns A and B* (Figure 1B). *Peg-pattern A* was designed for mice to run in a typical gait movement of a walk pattern. *Peg-pattern B* was designed to have a simpler pattern with twice as many pegs as *peg-pattern A*. We did not use any complex peg patterns because our preliminary experiments using DR KO mice showed that it was too difficult for DR KO mice to run on complex peg patterns.

After we tested mice in the rota-rod task, we set a normal breeding period of 1–4 weeks before testing the mice in the Step-Wheel task. We modified task procedures from the previous study (Kitsukawa et al., 2011) as follows. After 2 days of limited water access, we set pretrial sessions of 3 consecutive days, in which mice were allowed to drink water from the spout freely in the stationary wheel for 5 min to habituate themselves to the apparatus and for them to learn the location of the spout. In the pretrial sessions, we set the peg pattern to *peg-pattern B*. Between the groups of sessions, we set 1 day for rest, in which the mice were allowed to drink water for 4 min in their home cages. Next in the first test of the Step-Wheel task, turning speed of the wheel was initially set at 2 turns/min with *peg-pattern A*. In a particular group of sessions, the experimental conditions (peg pattern, turning speed, and number of turns of the wheel in one trial) were not changed. One trial was set for approximately 3 min. Thirty minutes after the end of a trial, we gave the mice additional water for 2 min in their home cages to maintain their health conditions. The first test consisted of sessions which consisted of 14 days with one rest day between the 7th and 8th sessions. After the first test, we set a normal breeding period (drinking water, *at libitum*) of 2–4 weeks. Following this period, we started the second test, in which we examined the motor ability of the mice at various speeds of the wheel with *peg-pattern B*. This test consisted of five consecutive daily sessions. The turning speed was adjusted to gradually increase (2, 3, 4, 5, 6, 7.5, and 10 turns/min in each group of sessions).

To evaluate the performance of mice in the Step-Wheel task, we used three different indices, namely *Touch Time, Water On Time*, and *Turn Stop*. *Touch Time* is the time during which the mice were in touch with the floorboard lying under the wheel, which was detected by the touch sensor (Figure 1A). Thus, *Touch Time* indicates the time that the animal was not on the pegs properly and touched the floorboard. *Water On Time* is the duration that mice were able to run sufficiently close to the spout, which was detected by *photobeam A* (Figure 1A). The data on *Touch Time* and *Water On Time* are presented as percentages of the duration of one trial. *Turn Stop* indicates the number of times that mice stopped turning the wheel. The wheel is designed to automatically stop when a mouse is at the most downstream in the wheel, at which the mouse is detected by *photobeam B* (Figure 1A).

### Data analysis and statistics

The data on spontaneous motor activity and food and water intake were analyzed by One-Way ANOVA followed by Tukey's test. The data from the rota-rod and Step-Wheel tasks were analyzed using the non-parametric Kruskal–Wallis test followed by the multiple comparison test (Mann–Whitney *U*-test) to compare the phenotypes of the mice of three genotypes, or using Dunnett test (followed by multiple comparison test) to compare the data obtained on the 1st day with those obtained on each of the following days within the same genotype.

## Results

### Characteristics of spontaneous motor activity

As reported previously, congenic D1R KO (McNamara et al., 2003) and D2R KO mice (Kelly et al., 1998; Clifford et al., 2001; Kim et al., 2010) exhibit growth retardation and small body weight compared with WT mice until the young adult stage. In this study, D1R and D2R KO mice grew sufficiently to carry out the following experiments comparable to WT mice (D1R KO, 27.48 ± 0.76 g, *n* = 4; D2R KO, 31.58 ± 1.35 g, *n* = 8; WT mice, 30.11 ± 1.33 g, *n* = 8). The exact reasons for the difference of body weight of D1 and D2 KO mice between the above reports and our experiments are unknown, but we suppose that we waited to use more matured D1R and D2R KO mice to gain enough weights comparable to WT mice.

**Figure 2:**
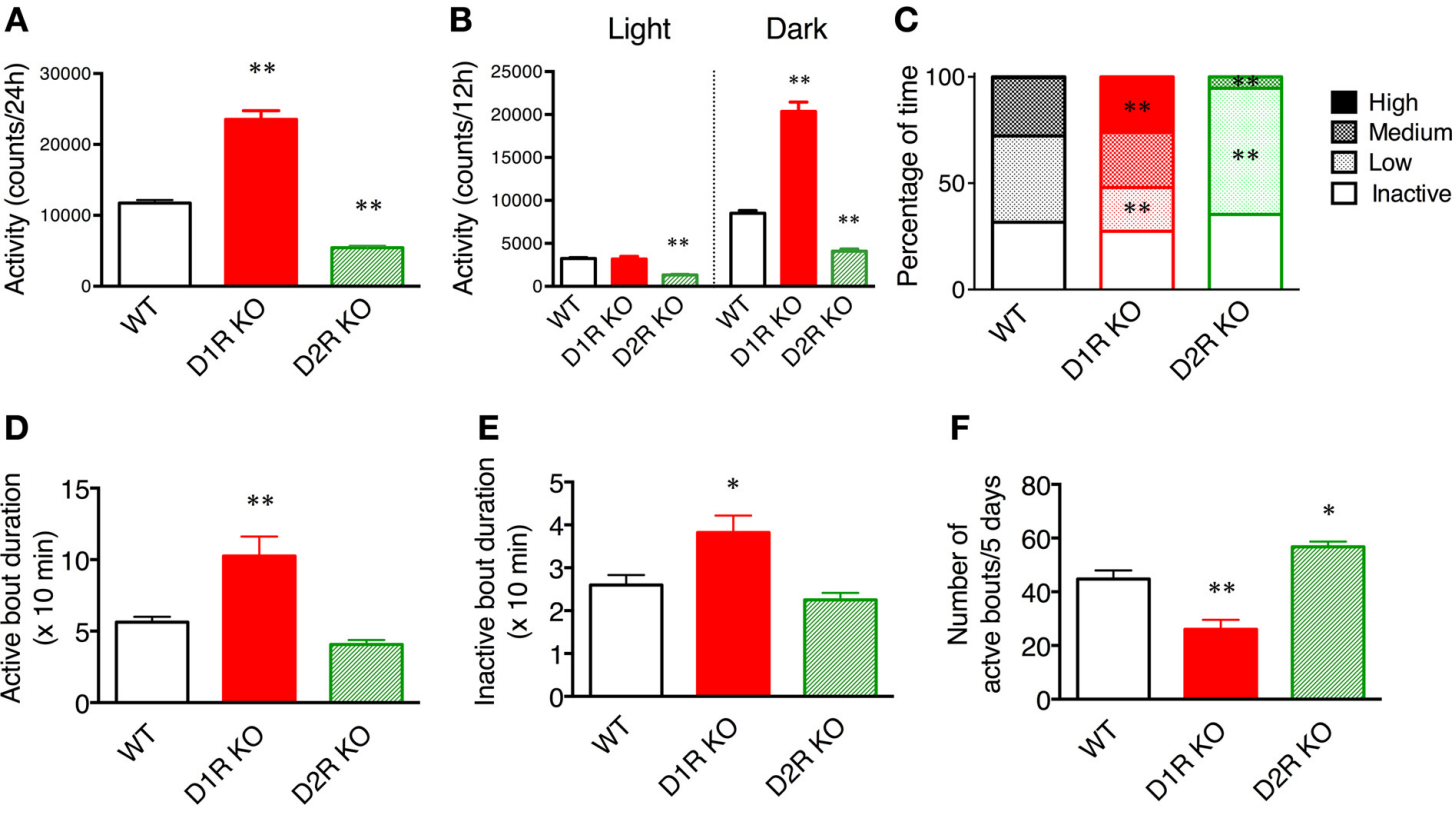
**Spontaneous motor activity of mice in home cages**. The home cage motor activity of WT (*n* = 8), D1R KO (*n* = 4), and D2R KO (*n* = 8) mice was monitored continuously for 5 days in a 12 h light/dark cycle. **(A)** Activity over 24 h. **(B)** Activity in 12 h light/dark phase. **(C)** Activity states in the dark phase were classified as follows on the basis of the numbers of movements: inactive state (0–9 counts/10 min), low- (10–199 counts/10 min), medium- (200–499 counts/10 min), high- (over 500 counts/10 min) active states. The percentage of time spent in each state is shown. **(D–F)**. The activity states in the dark phase were classified as the active state (over 10 counts/10 min) and inactive state (0–9 counts/10 min). **(D)** The durations of active bout duration, **(E)** the time of inactive bout duration, and **(F)** the number of active bouts per 5 days were then calculated. The values are mean ± s.e.m. ^*^p < 0.05, ^**^p < 0.01, vs. WT (One-Way ANOVA, Tukey's test).

We first examined spontaneous motor activity and both food and water intakes of D1R, D2R KO, and WT mice in the home cage environment for 5 consecutive days. The 24 h cumulative movements counted showed that D1R KO mice were more active (*p* < 0.01) whereas D2R KO mice were less active (*p* < 0.01) than WT mice (Figure 2A). These findings were consistent with previously reported open field analysis using congenic KO mice (Kelly et al., 1998; McNamara et al., 2003; Holmes et al., 2004). As indicated by a larger error bar, motor activity varied widely among individual D1R KO mice. Such variability has also been observed by Wall et al. (2011) as well. We omitted the data of three D1R KO mice that consumed only small amounts of food and water, because they were unable to continue the tasks. We examined in more detail the daily activities of mice in the following experiments.

To elucidate whether these KO mice have normal diurnal rhythms, we analyzed their motor activity during the light and dark phases. As similarly observed in WT mice, the motor activity in the dark phase was higher than that in the light phase in both KO mice (Figure 2B), indicating that both types of KO mice have normal diurnal rhythms. The activity of D1R KO mice was significantly higher than that of WT mice only during the dark phase (*p* < 0.01). The activity of D2R KO mice was significantly lower than that of WT mice in both the light and dark phases (*p* < 0.01). These findings showed that the hyperactivity of D1R KO mice came from their increased dark phase activity, whereas the hypoactivity of D2R KO mice came from their overall decreased daily activity.

To clarify the activity pattern in the dark phase, we carried out a detailed analysis based on activity count data per 10 min (Figures 2C–F). We classified activity states as follows: the inactive state, low-, medium-, and high-active states. Then, the percentage of time spent in each state was calculated (Figure 2C). Interestingly, both the D1R KO and D2R KO mice exhibited percentages of time in the inactive state comparable to that of WT mice. However, regarding the active state, the D1R KO mice spent a significantly higher percentage of time in the high-activity state than the WT mice (*p* < 0.01), and the D2R KO mice spent a significantly higher percentage of time in the low-activity state than the WT mice (*p* < 0.01). Because the active state of the mice was fragmented, we analyzed the duration and number of bouts of activity (Figures 2D–F). The duration of bouts of active and inactive states of the D1R KO mice were significantly longer than those of the WT mice (*p* < 0.01 and *p* < 0.05, respectively) and the number of active bouts was reasonably smaller in the D1R KO mice than in the WT mice (*p* < 0.01). On the other hand, the duration of active and inactive bouts of D2R KO mice tended to be shorter than those of the WT mice, although these differences were not statistically significant. The number of the active bouts was significantly greater in the D2R KO mice than in the WT mice (*p* < 0.05).

### Food and water intakes

**Figure 3:**
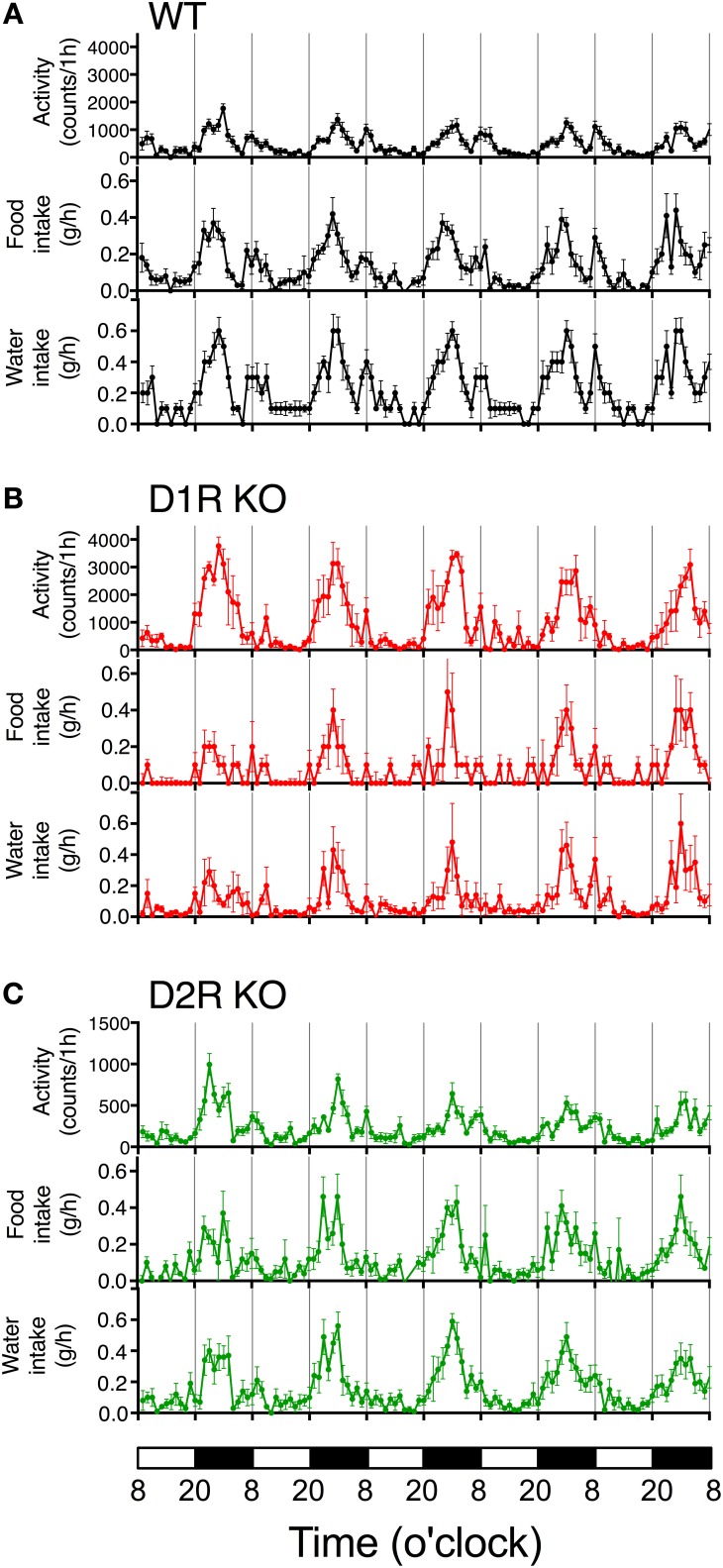
**Time courses of motor activity, intake, and water intake**. The activity counts and food and water intake profiles of **(A)** WT, **(B)** D1R KO, and **(C)** D2R KO mice are plotted against time (1 h time bins). The lower white and black bars represent the 12 h light and dark phase. The plotted values are mean ± s.e.m.

**Figure 4:**
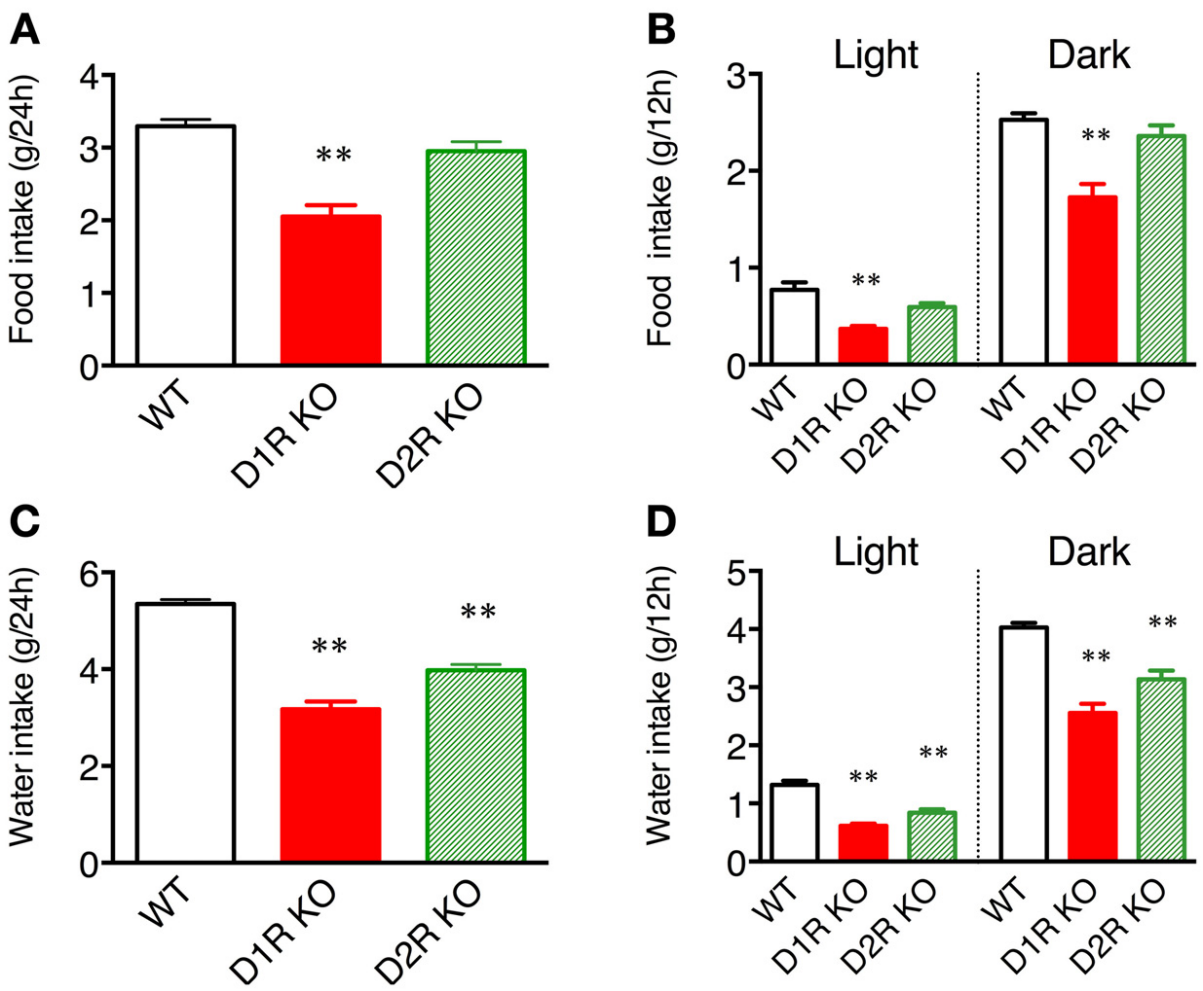
**Food and water intakes in home cages. (A)** Food intake over 24 h or **(B)** in 12 h light and dark phases, and **(C)** water intake over 24 h or **(D)** in 12 h light and dark phases are represented as group mean ± s.e.m. ^**^p < 0.01 vs. WT (One-Way ANOVA, Tukey's test).

Some of the behaviors accounting for the activity of mice are eating and drinking. Therefore, mean motor activity, food intake, and water intake were plotted against time (Figure 3). In all the genotypes, the time course of food and water intakes followed a similar trend to that of activity: a large peak appeared in the first half of the active phase and a small peak appeared at the end of the active phase. These findings indicate that both the D1R KO and D2R KO mice had normal rhythms of activity and food/water intakes. However, the D1R KO mice showed significantly smaller food and water intakes than the WT mice in spite of their excessively high activity (*p* < 0.01 in 24 or 12 h analysis of food and water intake, Figure 4). For the D2R KO mice, no significant difference in food intake was observed (*p* > 0.05) though they showed significantly smaller water intake than the WT mice (*p* < 0.01, Figure 4).

### Rota-rod test

**Figure 5:**
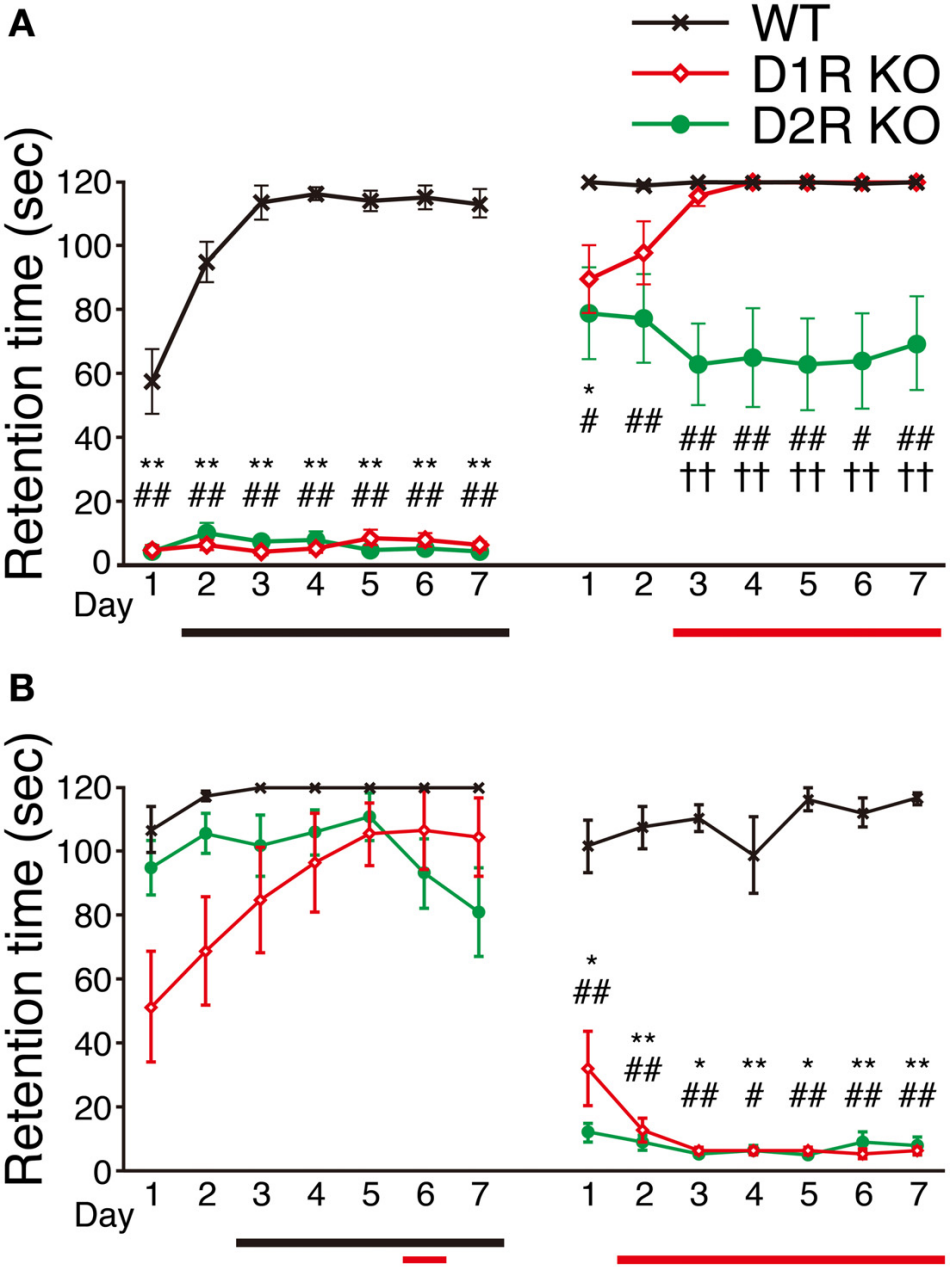
**Performance in rota-rod task**. The mice of each genotype were divided into two groups, **(A)** the fast-slow (F-S) group (fast, 15 rpm; slow, 5 rpm) and **(B)** the slow-fast (S-F) group. All the mice performed three trials per day. The retention time (sec) is represented as the mean ± s.e.m. of these three trials. WT mice (black crosses, F-S, *n* = 8, S-F, *n* = 5), D1R KO mice (red diamonds, *n* = 9 for each group), and D2R KO (green circles, *n* = 10, for each group). The maximum time of one trial was 120 sec. Significant differences between genotypes were found on days marked by ^*^p < 0.05, ^**^p < 0.01 for D1R KO vs. WT mice; ^#^*p* < 0.05, ^##^*p* < 0.01 for D2R KO vs. WT mice; ^††^*p* < 0.01, for D1R KO vs. D2R KO mice (Kruskal–Wallis test). Significant improvement of performances compared with the 1st day was found on days marked by horizontal bars shown under the graph (*p* < 0.05, Dunnett test: WT, black; D1R KO, red; D2R KO, green).

We next examined the motor ability of the mice with the rota-rod task. Most of the mice of three genotypes were able to stay for the maximum time (120 s) on the stationary rod in the presessions (data not shown). The mice of each genotype were divided randomly into two groups, the fast-slow (F-S) group and slow-fast (S-F) groups. The F-S group was exposed to a fast rotation in the first group of sessions and to a slow rotation in the second group of sessions. The S-F group was exposed conversely. First, we examined the mice of the F-S group at 15 rpm (Figure 5A), but both the D1R and D2R KO mice could not stay on the rod and showed no improvement. There were significant differences in performance between the KO mice and WT mice throughout the first group of sessions (*p* < 0.01). The performance of WT mice improved significantly (after the 2nd day, *p* < 0.05 compared with the 1st day). In the next group of sessions, the performance was measured at 5 rpm. Both the D1R and D2R KO mice could stay on the rod and were locomotive. On the 1st day of the second group of sessions, the retention time of both KO mice were significantly lower than those of the WT mice (*p* < 0.05). The D1R KO mice showed improvement (after the 3rd day, *p* < 0.05 compared with the 1st day) and the significant difference between the D1R KO and WT mice disappeared after the 2nd day. On the other hand the D2R KO mice showed a significantly lower performance throughout the second group of sessions than the WT mice (*p* < 0.05), and they did not improve. There were significant differences in performance between the D1R and D2R KO mice after the 3rd day of the second group of sessions (*p* < 0.01).

Next, we examined the mice of the S-F group by reversing the order of turning speed: first, in the slow and then fast turning (Figure 5B). In the sessions with slow rotation, the D1R KO mice showed improved performance. A significant difference in performance was observed between the 1st day and the 6th day (*p* < 0.05; p values on the 5th and 7th days were *p* = 0.053 and *p* = 0.059, respectively). On the other hand, D2R mice did not show any improvement. The WT mice showed improvement in the slow rotation sessions (after the 3rd day, *p* < 0.05 compared with the 1st day). There was no significant difference in the retention time among the WT, D1R, and D2R KO mice on any day of the slow rotation sessions. In the next sessions of the S-F group (15 rpm), both the KO mice exhibited significantly lower performance than the WT mice throughout the second group of sessions, which was similar to the finding in the first sessions of the F-S group. The WT mice of the S-F group showed no improvement in the fast rotation whereas the mice in the F-S group showed improvement. This difference may be attributed to the fact that the mice in the S-F group had already experienced the rota-rod task at a low speed.

Although the D1R KO and WT mice of both the F-S and S-F groups showed similar trends, the D2R KO mice of these two groups showed a difference in the slow rotation sessions. We will discuss the findings in detail later.

### Improvement of performance in step-wheel task

**Figure 6:**
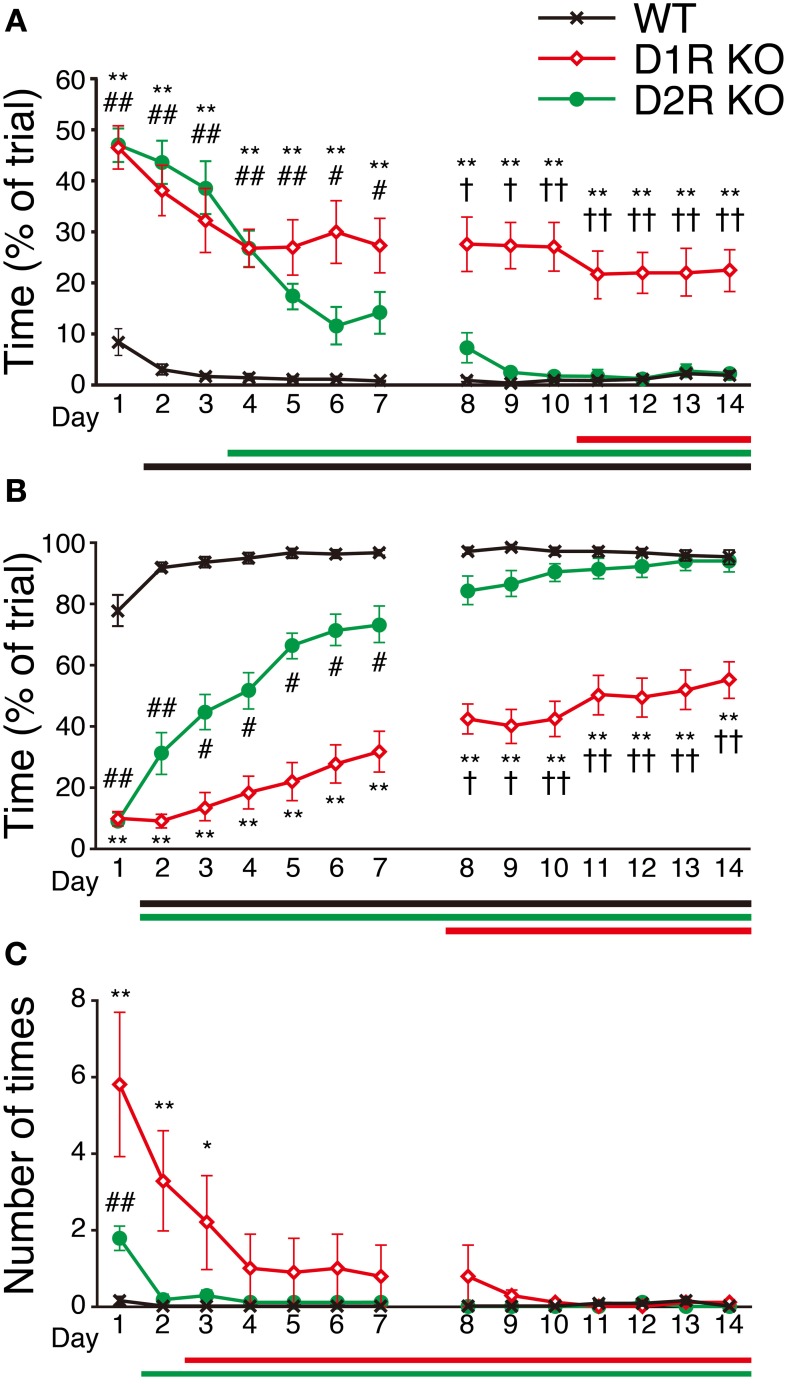
**Performance in Step-Wheel task. (A)** *Touch Time* (% of trial), **(B)** *Water On Time* (% of trial), and **(C)** number of *Turn Stops* are represented as group means ± s.e.m. Significantly differences in performance between genotypes were found on days marked by ^*^p < 0.05, ^**^p < 0.01 for D1R KO vs. WT mice; ^#^*p* < 0.05, ^##^*p* < 0.01 for D2R KO vs. WT mice; ^†^*p* < 0.05, ^††^*p* < 0.01 for D1R KO vs. D2R KO mice (Kruskal–Wallis test). Significant improvement of performances compared with that on the 1st day was found on days marked by horizontal bars shown under the graphs (*p* < 0.05, Dunnett test; WT, black; D1R KO, red; D2R KO, green).

To examine the motor ability and motor improvement of each genotype, we conducted the Step-Wheel task. Mice were first trained with *peg-pattern A* for 14 days. We used three indices to estimate their performance, *Touch Time* (Figure 6A), *Water On Time* (Figure 6B) and *Turn Stop* (Figure 6C), as described in Materials and Methods. In the Step-Wheel task, both genotypes of KO mice showed improved performance but they needed a longer time to reach the plateau than the WT mice (Figures 6A,B). Although the D1R KO mice showed improvement, the plateau of their performance was lower than those of the WT and D2R KO mice (*p* < 0.01). In comparison with the performance of the D1R KO mice on the 1st day, statistically significant differences in *Touch Time* and *Water On Time* were observed after the 11th and 8th days, respectively (*p* < 0.05). The performances of D1R KO mice in *Touch Time* and *Water On Time* were significantly lower than those of the WT mice on all the days (*p* < 0.01), and also lower than those of D2R KO mice in the late phase of *Touch Time* and *Water On Time* (after the 8th day, *p* < 0.05). D2R KO mice showed significant improvement and were able to finally reach the same level of performance as the WT mice did, which was in sharp contrast to the performance of the D1R KO mice. Although the performance of the D2R KO mice was much lower than that of the WT mice in the early phase in terms of *Touch Time* and *Water On Time* (during the first 7 days, *p* < 0.05 in both *Touch Time* and *Water On Time*), it became comparable to that of WT mice in the late phase. In comparison with the performance of the D2R KO mice on the 1st day, significant differences in *Touch Time* and *Water On Time* were observed after the 4th and 2nd days, respectively (*p* < 0.05). The WT mice could almost reach their peak performance on the 2nd day, as shown by the significant differences in both *Touch Time* and *Water On Time* from those on the 1st day (*p* < 0.05).

### Performance at various speed in step-wheel task

**Figure 7:**
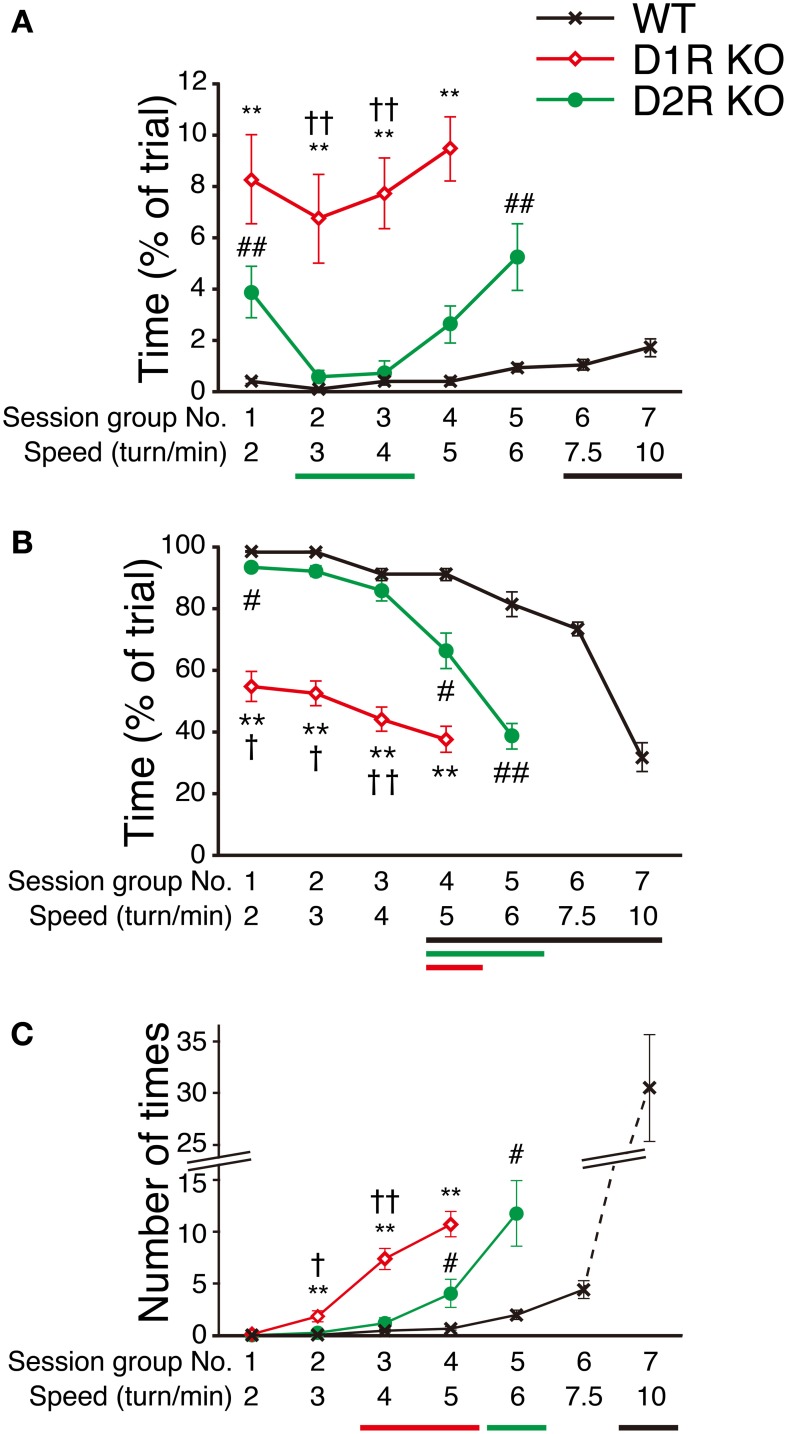
**Performance at various speeds in Step-Wheel Task. (A)** *Touch Time* (% of trial), **(B)** *Water On Time* (% of trial), and **(C)** number of *Turn Stops* at various turning speeds are represented as group means ± s.e.m. over 5 trials in each group of sessions. The turning speed was set to 2, 3, 4, 5, 6, 7.5, and 10 turns/min, from the 1st to 7th group of sessions, respectively. WT mice (black crosses, *n* = 13), D1R KO mice (red diamonds, *n* = 10), D2R KO mice (green circles, *n* = 10). Significantly different performances between genotypes were found in the group of sessions marked by ^**^p < 0.01 for D1R KO mice vs. WT mice; ^#^*p* < 0.05, ^##^*p* < 0.01 for D2R KO mice vs. WT mice; ^†^*p* < 0.01, ^††^*p* < 0.01 for D1R KO mice vs. D2R KO mice (Kruskal–Wallis test). Significant differences from the 1st group of sessions were found in the groups of sessions marked by horizontal bars shown under the graphs (*p* < 0.05, Dunnett test; WT, black; D1R KO, red; D2R KO, green).

To clarify the motor ability of each genotype in more detail, we next examined how fast mice could run in the Step-Wheel system by gradually increasing the speed (seven different speeds, 2, 3, 4, 5, 6, 7.5, and 10 turns/min for each group of sessions, from the 1st to 7th group of sessions) with a simple peg pattern: *peg-pattern B* (Figure 1B). The mice were trained for 5 days at each speed. The average of the scores on these 5 days was used for the comparison among genotypes (Figure 7). The D1R KO mice showed a significantly lower performance in *Touch Time* and *Water On Time* than the WT mice from the 1st to 4th group of sessions (*p* < 0.01). In the 5th group of sessions (6 turns/min), the D1R KO mice were not able to keep pace with the speed. Thus, we considered that the speed in the 4th group of sessions (5 turns/min) was the limit for the D1R KO mice. The D2R KO mice showed a longer *Touch Time* than WT mice on the 1st group of sessions although no and much less differences of the value were found in *Turn Stops* and *Water On Time*, respectively, but showed similar values in all the three indices on the 2nd and 3rd sessions. The performance of the D2R KO mice became significantly worse (*p* < 0.05 compared with the 1st group of sessions) after the 4th group of sessions in *Water On Time*, in the 5th group of sessions (6 turns/min) in *Turn Stops*. We considered that this speed (6 turns/min) was the maximum for the D2R KO mice. The WT mice showed a high performance, but their performance gradually became worse with the progression of the group of sessions. Finally, in the 7th group of sessions (10 turns/min) their performance suddenly dropped in *Water On Time* and *Turn Stops*; we considered that this speed was the maximum for the WT mice.

These results demonstrated that the Step-Wheel System enables the clear discrimination of performance levels among the WT, D1R KO, and D2R KO mice.

## Discussion

There have been several studies of the motor activity of D1R and D2R KO mice, but the results for D1R KO mice are incongruent (Drago et al., 1994; Xu et al., 1994a,b; Smith et al., 1998; Centonze et al., 2003; Tran et al., 2005, 2008). The possible reasons for these discrepancies are the differences in the genetic background among various line of KO mice and experimental procedure (Kelly et al., 1998; Fowler et al., 2002; McNamara et al., 2003; Holmes et al., 2004; Waddington et al., 2005). Therefore, in this study we used N10 congenic KO mice (C57BL/6, backcrossed over 10 generations) and compared their home cage activity as normal spontaneous motor activity for 5 consecutive days. This method of measuring activity is less influenced by the variation of external factors. Furthermore, we compared the motor ability using the rota-rod and Step-Wheel tasks of the N10 congenic D1R and D2R KO mice and detected significant differences among the three genetic types of mice.

### Spontaneous motor activity

We measured the spontaneous motor activities of D1R KO, D2R KO and WT mice using the 24 h monitoring system (Figure 2). The results showed that D1R KO mice were higher 24 h activity levels (hyperactivity) than WT mice whereas D2R KO mice were lower 24 h activity levels (hypoactivity) than WT mice in the home cage environment. These results in congenic mice were consistent with previous reports regarding spontaneous motor activity reported by other groups despite of the difference of the task or monitoring time (Kelly et al., 1998; McNamara et al., 2003; Wall et al., 2011). To further characterize their behavior, we analyzed the activity pattern. D1R KO mice showed an increased activity only in the dark phase (Figures 2B, 3). A detailed analysis revealed that the D1R KO mice moved actively and the durations of active and inactive bouts were long, although the total time in the inactive (resting) state was comparable to that in WT mice (Figure 2). In our recorded video image, D1R KO mice exhibited stereotypical behavior during the active state: they repeatedly hung on the lid and descended to the floor. This abnormal behavior and reduced food and water intakes in the D1R KO mice suggest that their hyperactivity is not due to increased activities associated with taking food and water. It seems that excess motor activity was not well controlled once D1R KO mice became active. Previous studies indicated that D1R KO mice could not take food and water effectively because of a deficit in fine motor control (Drago et al., 1994; Xu et al., 1994a,b; Holmes et al., 2004), which is consistent with the present observations. Other deficits in the reward system could also affect the activity pattern in D1R KO mice. On the other hand, in the D2R KO mice, decreased motor activity was observed in both the light and dark phases, and the time course patterns showed low activities at all times (Figure 3). These mice showed a lower activity than WT mice. Nonetheless, their food intake was not significantly different from that in WT mice. It is conceivable that hypoactivity in D2R KO mice may be caused by general motor dysfunction rather than the reward system. For better understanding, we next examined the motor ability of D1R and D2R KO mice using the rota-rod and Step-Wheel tasks (Kitsukawa et al., 2011).

### Motor performance in rota-rod and step-wheel task

D1R and D2R KO mice were able to stay on the stationary rod (data not shown) and slowly rotating rod to some extent (Figure 5, Supplementary Figure 1). These findings indicated that both KO mice have no severe motor dysfunctions such as ataxia or catalepsy, which were reported previously (Kelly et al., 1998; Clifford et al., 2001; Tran et al., 2005, 2008). Nonetheless, being consistent with the studies that reported D1R KO (Drago et al., 1994; Xu et al., 1994a,b; Holmes et al., 2004) and D2R KO mice (Baik et al., 1995; Kelly et al., 1998; Fowler et al., 2002) showed poorer motor function than WT mice, the results of fast rotation in the rota-rod task (Figure 5) indicated a much worse motor ability of D1R and D2R KO mice than of WT mice. Comparison of the motor ability of D1R KO mice with that of D2R KO mice in the slow sessions showed conflicting results for the D2R KO mice regardless of whether the slow sessions was carried out firstly or secondly. In the 1st group of sessions, the S-F group of D2R KO mice achieved well (Figure 5B), whereas in the 2nd group of sessions, the F-S group showed a somewhat worse performance than that of the other two genotypes (Figure 5A). Analysis of the data of individual D2R KO mice (Supplementary Figures 1C,F) revealed that these mice showed differences in performance throughout the sessions. Some mice were able to stay longer on the rota-rod while others were not, and most of them showed no improvement. Considering these results, the score of D2R KO mice at a low speed may be dependent not only on motor ability but also other factors. We speculate that this may be attributed to the task features, as will be discussed later, or previous failed trials at a fast rotation may have impaired the task performance of these mice even in a low speed. If this is true for D2R KO mice, it may be difficult to evaluate their motor ability in the rota-rod task. Indeed, from these results of the rota-rod task, it was unclear which showed better performance between the D1R KO and D2R KO mice and whether the D2R KO mice could improve their performance or not, whereas the D1R KO mice showed improvement. Previous studies showed that D2R KO mice could improve their performance in the rota-rod task although it may be poor in the beginning (Kelly et al., 1998; Fowler et al., 2002). Therefore, we next examined the motor ability of D1R and D2R KO mice using the Step-Wheel System.

The results of the Step-Wheel task clearly demonstrated that the motor ability among the mice of three genotypes was in the order of WT, D2R KO, and D1R KO mice (Figures 6, 7), which also indicated the usefulness of this system. The results of the Step-Wheel task (Figure 6) showed that the mice of all of the three genotypes could improve their performance and that the time courses of their performances of improvement differed. D1R KO mice could improve their performance but their plateau levels were much lower than those of the other two genotypes. We observed that in the late phase they still made many missteps and did not grip the pegs, thereby putting their legs on the floorboard, and they could not keep the position close to the water spout. We speculated that the D1R KO mice have low motor ability for fine motor skills for the Step-Wheel task. Furthermore, the D1R KO mice appeared to have a lower motivation to drink water. When the wheel was not turning (at the start of the trial or at restart after *Turn Stops*), D1R KO mice took much time to approach the spout, whereas the mice of the other two genotypes did it quickly. The D1R KO mice took a smaller volume of water and a shorter time to drink in the stationary wheel than the other two genotypes (Supplementary Figure 2). Tran et al. (2005) previously indicated that D1R KO mice have some deficits in the reward task. However, as the Step-Wheel task processed, the D1R KO mice improved slightly in terms of *Touch Time* and *Water On Time*. Therefore, we interpreted these findings as D1R KO mice having lower motivation to drink water and poorer motor ability than the mice of the other genotypes.

The WT mice reached almost the plateau of their performance by the 2nd day (Figure 6), which indicated that the condition (peg pattern and turn speed) was very easy for the WT mice. Indeed, the WT mice were able to run from the first test (e.g., 3–4 turns/min, Supplementary Figure 3) at the speed that neither the D1R KO nor D2R KO mice were able to run. These findings also indicated that both the D1R and D2R KO mice have poorer motor ability than the WT mice.

### Behavioral features in rota-rod and step-wheel tasks

The rota-rod task is wildly used to evaluate the motor function and skill learning of rodents and requires animals to have good body balance for their locomotion. The Step-Wheel system is designed for assessing limb coordination, locomotor adaptation to different running speeds, and stepping patterns of mice (Kitsukawa et al., 2011). These two behavioral tasks differ as follows. (1) The required motor abilities are different. The rota-rod task requires mice to balance themselves on the flat surface of the circular rod, whereas the Step-Wheel task requires mice to grip the pegs and adjust their legs to spatiotemporal timing. The Step-Wheel task may require a more precise motor control than the rota-rod task. (2) The incentives for mice differed. In the rota-rod task, mice should try and balance themselves to avoid dropping; therefore, this task is an aversive task. The Step-Wheel task requires a mouse to keep pace with the turning speed to keep on drinking water as the reward. Hikida et al. (2010) reported that the direct pathway, in which D1Rs are expressed, is critical for reward learning, whereas the indirect pathway, in which D2Rs are expressed, is critical for aversive learning. Their finding may be related to our results, which showed mismatches between the D1R KO mice and the Step-Wheel (reward-driven) task and between the D2R KO mice and the rota-rod (aversive) task.

Water intake of D2R KO mice for 24 h in home cage was lower than that for WT mice (Figure 4). In addition, Elmer et al. (2002) reports the decreased water intake in D2R KO mice in lever pressing responses compared to WT mice whereas Risinger et al. (2000) reports that there is no significant difference in water intake between D2R KO and WT mice. Therefore, the water intake of D2R KO mice could be variable depending on the conditions. In any case, as a matter of fact, in the presessions of Step-Wheel task there was essentially no significant difference between D2R KO and WT mice or even higher at 5 min in day 1 (Supplementary Figure 2). Therefore, we consider that D2R KO mice have at least enough motivation to drink water at low speeds in the Step-Wheel task. Soto et al. (2011) report that, in lever press and nose poke tasks, reinforcing effectiveness is decreased in D2R KO mice compared to WT mice. Risinger et al. (2000) and Elmer et al. (2002) also report that D2R KO mice show lower lever responses in increased dose of morphine and ethanol than WT mice. Taken together, we interpret that D2R KO mice have enough motivation for drinking water to perform the Step-Wheel task at low speeds and perform the task moderately well compared to WT mice at the low speed (Figure 7). At high speeds (Figure 7, more than 7.5 turns/min), D2R KO mice no longer performed the Step-Wheel task at all. This default of the task at the high speeds in D2R KO mice was probably due to the decreased reinforcing effectiveness because of dopamine D2R deletion (Soto et al., 2011).

In this study, the difference in ultimate motor ability between the D1R KO and the D2R KO mice in the rota-rod task was unclear, although we did not try the accelerating rota-rod task (Wall et al., 2011; Durieux et al., 2012). Wall et al. (2011) reported that D1R KO mice exhibited little improvement in the accelerating rota-rod task, but the maximum speed at which the D1R KO mice could keep pace with was about 12–15 rpm. This speed was similar to that in our results of the rota-rod task at a high speed (Figure 5). Taken together, the report by Wall et al. and our results demonstrate that D1R KO mice are able to improve at a low speed but not at a high speed in the rota-rod task.

### The difference in three behaviors between D1R and D2R KO and the implications

We examined the behaviors of congenic D1R and D2R KO mice by 24 h spontaneous activity monitoring and using the rota-rod and Step-Wheel tasks. These three behavioral experiments clearly distinguished the differences among the mice of these genotypes.

Durieux et al. (2012) researched the functions of the direct and indirect pathways by selective ablation of D1R-expressing striatonigral MSNs and D2R-expressing striatopallidal MSNs (D1R MSNs and D2R MSNs, respectively) with spatial resolution in the dorsomedial striatum (DMS) or dorsolateral striatum (DLS). They reported that the ablation of D1R MSNs in DMS and the entire striatum reduced locomotion in the open field. These findings are the reverse of those in D1R KO mice. The hyperactivity of D1R KO mice may be due to the lack of D1R in the developmental stage or other regions besides the striatum in the adult stage. Xu et al. (1994b) suggested that the hyperactivity of D1R KO mice is due to the reduction of D1R-mediated dopamine transmission in other limbic and cortical areas, resulting in the relief of a normal inhibitory influence by these structures. We observed the D1R KO mice repeating stereotyped behavior. It remains to be elucidated how altered neural pathways in D1R KO mice induce this behavior. In contrast, the ablation of D2R MSNs in DMS and the entire striatum increased locomotion (Durieux et al., 2012). Sano et al. (2003) reported that conditional ablation of striatal D2R MSNs caused hyperactivity in the open field. Our study showed the hypoactivity of D2R KO mice. All of these results are consistent with the functions of the direct and indirect pathways and D2R MSNs, as Murer et al. (2000) suggested that hyperactivity in the subthalamic nucleus causes the hypoactivity of D2R KO mice.

Durieux et al. (2012) also reported the effects of local ablation on the motor control and skill learning in the accelerating rota-rod tasks. The ablation of D1R MSNs in DLS or the entire striatum caused poor performance until the late phase, whereas the ablation of D2R MSNs in DMS or the entire striatum impaired the performance only in the initial phase. These findings are in good agreement with our results and we speculate as follows. The main defect of motor ability in D1R KO mice is likely due to the dysfunction of D1R MSNs in DLS. On the other hand, slower improvement of D2R KO mice than WT mice in the Step-Wheel task is due to the dysfunction of D2R MSNs in DMS. One important difference is that the ablation of D2R MSNs in either DMS or the entire striatum does not affect the late-phase performance (Durieux et al., 2012), whereas the D2R KO mice showed poorer learning than the WT mice in the Step-Wheel task (Figure 6). Previous studies indicated that dopamine in the motor cortex plays certain functions for motor skill learning in the precision forelimb reaching tasks (Molina-Luna et al., 2008; Hosp et al., 2011). The difference in the late-phase of motor learning between mice with ablated D2R MSNs and general D2R KO mice is one of the interesting points that remain to be clarified in future studies.

### Conflict of interest statement

The authors declare that the research was conducted in the absence of any commercial or financial relationships that could be construed as a potential conflict of interest.
