# Supplementary material for: Distinct motor impairments of dopamine D1 and D2 receptor knockout mice revealed by three types of motor behavior
Source: Front Integr Neurosci. 2014 Jul 15;8:56. doi: 10.3389/fnint.2014.00056 (PMC4097398; doi:10.3389/fnint.2014.00056)
Supplement: Supplementary file 1 [file Presentation1.ZIP › Supplementary Material.pdf]

Supplementary Material

Distinct motor impairments of dopamine D1 and D2 receptor knockout mice revealed by three types of motor behaviors

Toru Nakamura[1,2]†, Asako Sato[3,4]†, Takashi Kitsukawa[1,5], Toshihiko Momiyama[6], Tetsuo Yamamori[1,2]* Toshikuni Sasaoka[3,4,7]*

[1]Division of Brain Biology, National Institute for Basic Biology, Okazaki, Japan
[2]Department of Basic Biology, Graduate University for Advanced Studies (SOKENDAI), Okazaki, Japan
[3]Laboarroty of Neurochemistry, National Institute for Basic Biology, Okazaki, Japan
[4]Department of Laboratory Animal Science, Kitasato University School of Medicine, Sagamihara, Japan
[5]KOKORO-Biology Group, Laboratories for Integrated Biology, Graduate School of Frontier Biosciences, Osaka University, Osaka, Japan
[6]Department of Pharmacology, Jikei University School of Medicine, Tokyo, Japan
[7]Department of Comparative and Experimental Medicine, Brain Research Institute, Niigata University, Niigata, Japan

† These authors equally contributed, * Correspondence: Toshikuni Sasaoka, Department of Comparative and Experimental Medicine, Brain Research Institute, Niigata University, 1-757 Asahimachi-dori, Chuo-ku, Niigata, Niigata 951-8585, Japan, Email: sasaoka@bri.niigata-u.ac.jp; Tetsuo Yamamori, Division of Brain Biology, National Institute for Basic Biology, Okazaki, Aichi 444-8585, Japan, Email: yamamori@nibb.ac.jp

Supplementary Figures

Supplementary Figure 1 | Performance of individual mice in rota-rod task.

(A, B, C) The Fast-slow (F-S) group (fast, 15 rpm; slow, 5 rpm) and **(D, E, F)** slow-fast (S-F) group performed three trials per day. Retention time (sec) is represented as the mean of these three trials. **(A, D)** WT (black, F-S, n = 8; S-F, n = 5), **(B, E)** D1R KO (red, n = 9 for each group) and **(C, F)** D2R KO (green, n = 10, for each group).

Supplementary Figure 2 | Water drinking behavior in stationary Step-Wheel system.

(A) Accumulative water volume and **(B)** drinking time were recorded every 30 sec up to 5 min per day. Data are represented as mean ± s.e.m. WT (black, n = 13), D1R KO (red, n = 10) and D2R KO (green, n = 10). The first 3 days were recorded as presession and the latter 3 days were after the Step-Wheel task. Significant differences between genotypes at 3 min (which was the same as the time of one trial of the Step-Wheel task) and at 5 min of each day are indicated by ** $p < 0.01$ for D1R KO vs WT mice; # $p < 0.05$, ## $p < 0.01$ for D2R KO vs WT mice; † $p < 0.05$, †† $p < 0.01$ for D1R KO vs D2R KO mice (Kruskal-Wallis test).

Supplementary Figure 3 | Performance of WT mice in Step-Wheel task at different speeds.

WT mice were subjected to the Step-Wheel task at two different speeds, 3 turns/min (light blue diamonds, n = 3) and 4 turns/min (black circles, n = 5) for 10 consecutive days using *peg-pattern A*. **(A)** *Touch Time*, **(B)** *Water On Time*, and **(C)** numbers of *Turn Stops* are represented as mean ± s.e.m. Significant improvement of performance compared with performance on the 1st day was found on days marked by horizontal bars shown under the graphs ($p < 0.05$, Dunnett test; 3 turns/min, light blue; 4 turns/min, black).
