# Supplementary figures and images for: Distinct motor impairments of dopamine D1 and D2 receptor knockout mice revealed by three types of motor behavior
Source: Front Integr Neurosci. 2014 Jul 15;8:56. doi: 10.3389/fnint.2014.00056 (PMC4097398; doi:10.3389/fnint.2014.00056)

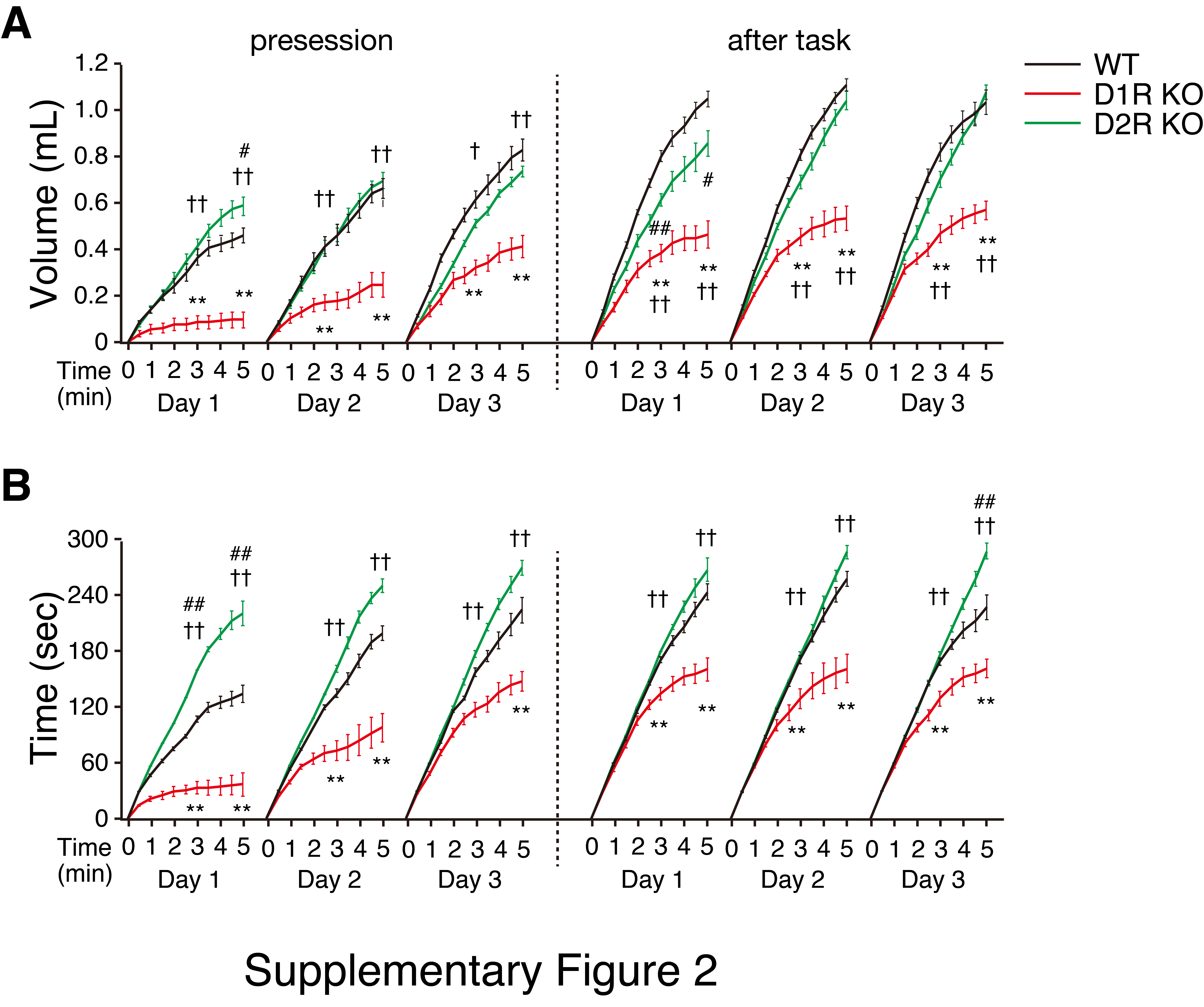

Supplement: Supplementary file 1 [file Presentation1.ZIP › Sup Fig 2.TIF]

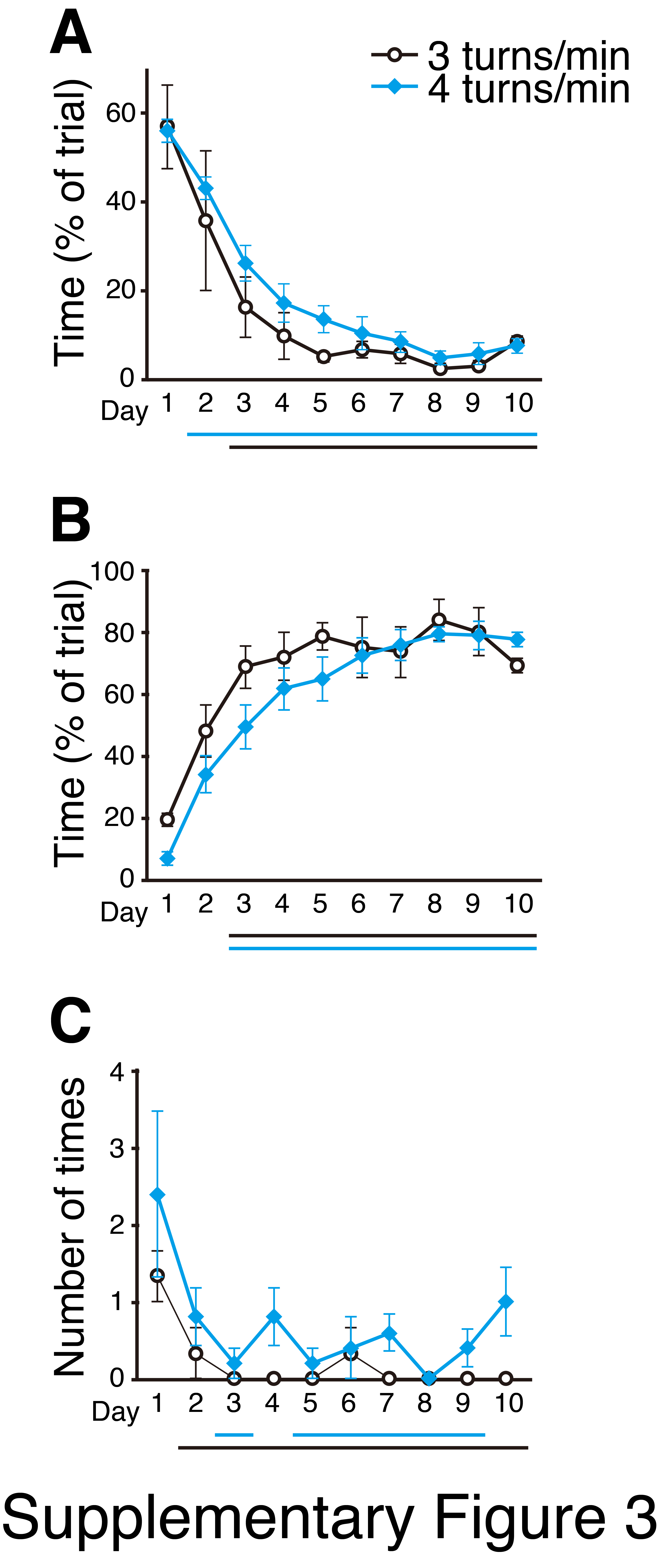

Supplement: Supplementary file 1 [file Presentation1.ZIP › Sup Fig 3.TIF]

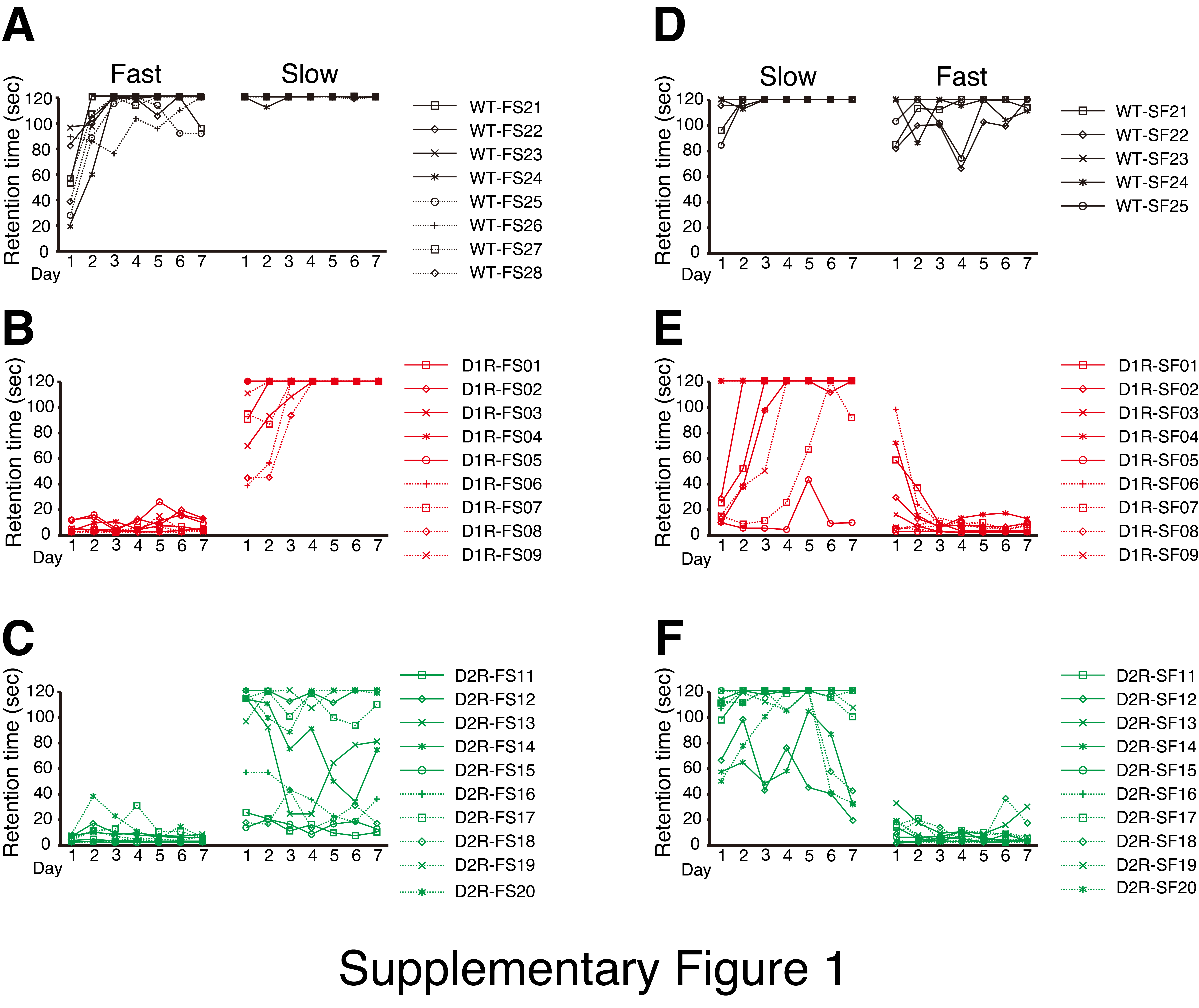

Supplement: Supplementary file 1 [file Presentation1.ZIP › Sup Fig 1.TIF]
